# Differential Mitochondrial Genome Expression of Four Skink Species Under High-Temperature Stress and Selection Pressure Analyses in Scincidae

**DOI:** 10.3390/ani15070999

**Published:** 2025-03-30

**Authors:** Xuxiang Wu, Lemei Zhan, Kenneth B. Storey, Jiayong Zhang, Danna Yu

**Affiliations:** 1 College of Life Sciences, Zhejiang Normal University, Jinhua 321004, China; 2 Department of Biology, Carleton University, Ottawa, ON K1S 5B6, Canada; 3 Key Lab of Wildlife Biotechnology, Conservation and Utilization of Zhejiang Province, Zhejiang Normal University, Jinhua 321004, China

**Keywords:** skink, high-temperature stress, selection pressure, *RT*-qPCR, mitochondrial genome expression

## Abstract

As ectotherms highly sensitive to environmental temperature fluctuations, skinks (a small lizard) are increasingly vulnerable to population instability due to global heatwaves. A clade model analysis of four Chinese skink species (*Plestiodon capito*, *Plestiodon chinensis*, *Sphenomorphus indicus*, and *Scincella modesta*) revealed positive selection acting on the ND6 gene in *Sp. indicus*. This species exhibits codon alterations in ND6, shifts its expression pathway and potentially decouples ND6 from high-temperature stress response mechanisms. To validate these findings, transcriptomic profiling was conducted to assess mitochondrial protein-coding gene (PCG) expression patterns under thermal stress. Using *RT*-qPCR, liver mitochondrial PCG transcript levels were compared between high-temperature (34 °C) and control (25 °C) groups in skink populations from distinct latitudes. Low-latitude species (*P. chinensis* and *Sc. modesta*) exhibited metabolic downregulation, characterized by a significant suppression of mitochondrial gene expression. Specifically, *P. chinensis* showed the downregulation of six mitochondrial genes (COII, COIII, ATP6, ND2, ND4, ND6) while upregulating one (ND1). By contrast, *Sc. modesta* showed the downregulation of nine genes (COI, COII, COIII, ATP8, ND1, ND3, ND4, ND4L, CYTB) and upregulated two (ND5, ND6). By contrast, high-latitude species exhibited divergent patterns: *P. capito* downregulated four genes (COI, COII, COIII, ND4L) and upregulated four others (ND1, ND2, ND3, ND4), whereas *Sp. indicus* downregulated six genes (COI, COII, ND2, ND3, ND4, ND4L) and upregulated one (ND5). These regulatory disparities suggest that low-latitude skinks have a greater capacity for metabolic depression to cope with chronic stress, whereas their high-latitude counterparts exhibit different adaptations. The findings provide valuable insights into assessing the adaptive potential of species in warming environments, particularly for ectotherms with limited thermoregulatory capacities.

## Simple Summary

The increasing occurrence of extreme heatwaves globally affects the stability of species populations and their distribution ranges. The present study shows that the ND6 gene of the skink, *Sphenomorphus indicus*, has undergone positive selection. We then compared the differences in mitochondrial genome expression levels under high-temperature stress among four skink species distributed across different latitudes: *Plestiodon capito*, *Plestiodon chinensis*, *Sphenomorphus indicus*, and *Scincella modesta*. Two metabolic strategies of mitochondrial genome expression that vary with latitude were identified.

## 1. Introduction

Against a backdrop of global warming and the increasing frequency of extreme climate events, the global average temperature continues to rise, and temperature fluctuations within small-scale regions have become more pronounced [1,2]. For skinks, which rely predominantly on environmental temperature for thermoregulation, this presents a considerable challenge, particularly in high-temperature environments [3,4]. Temperature variations can disrupt the inherent homeostasis of skinks and elicit their responses to adapt to new environmental temperatures. Therefore, the ongoing rapid increase in global temperatures is likely to change the strategies that skinks have evolved for adapting to environmental temperature changes over millions of years [5]. This inevitably results in a decline in the biodiversity of reptiles worldwide (www.iucnredlist.org, accessed on 14 November 2024). Skinks exhibit exceptionally high ecological value, which is manifested in their near-global distribution, high species richness, and remarkable interspecific diversity [6]. For instance, they display significant variation in body size [7], reproductive strategies (including both oviparous and viviparous modes) [8], and morphological adaptations such as reduced or absent limbs in certain species [9]. Their ecological and life-history traits also encompass extensive diversity, including thermal preferences, activity periods, social behavior, and dietary specialization [6]. Such multifaceted variation underscores their critical roles in ecosystem functioning. Consequently, global temperature changes may therefore drive population declines in skinks, potentially destabilizing ecosystem stability.

Skink populations distributed along latitudinal or altitudinal gradients may be subject to varying intensities and durations of environmental thermal stress, which could lead to differences in their evolutionary response mechanisms over the long term [10,11,12]. Consequently, under different levels of historical exposure, the adaptive mechanisms of species vary [13,14,15]. Species from regions with high temperatures tend to employ metabolic strategies that are better suited for coping with heat, whereas those that do not frequently encounter high temperatures may instead adopt a contrasting metabolic approach [16,17,18]. This is specifically reflected in the variation of mitochondrial PCGs or other genes associated with metabolism, where some genes may undergo selection, and there may also be variations in the expression levels of certain genes.

Mitochondria, as the energy factories of the cell, are intimately connected with the energy metabolism of organisms, particularly in close association with temperature factors [19,20]. They are also believed to have important indicative roles in biogeography and species ecology [21,22]. Through a deeper comprehension of the mechanisms by which species respond to climate, it becomes feasible to determine the distribution ranges of species populations, which is instrumental in conserving biodiversity and in proactively responding to climate change. The mitochondrial genome includes 13 PCGs that encode proteins that are integral parts of the mitochondrial respiratory complexes I–V, and they play a significant role in establishing the proton gradient that facilitates the generation of ATP [23,24]. Beyond their primary functions, mitochondria mediate signaling cascades, modulate cellular viability, regulate calcium ion balance, and participate in immunological processes [25,26,27]. Therefore, selecting mitochondrial genes as the focal subject of this study facilitates a deeper understanding of the relationship between energy metabolism variations and species’ geographical distribution.

The selection pressures exerted by climatic and geographical variations on mitochondrial genes, as well as the associated differences in gene expression levels, have been elucidated in previous studies. Lineage-specific selection pressures acting on mitochondrial DNA may arise from habitat heterogeneity or spatial segregation across biogeographical ranges. Evolutionary analyses employing codon-substitution models within maximum likelihood frameworks quantify adaptive evolution through *ω* (*dN/dS*) [28]. If *ω* < 1, it is considered that purifying selection has occurred, whereas if *ω* > 1, it is considered that positive selection has occurred. If *ω* = 1, then neutral selection has occurred [29]. Through selection pressure analysis of *Takydromus intermedius*, the present study shows that ATP6, ATP8, and ND3 underwent positive selection and were most strongly correlated with climatic variables [30]. Phylogenetic selection analyses across 15 *Phrynocephalus* taxa inhabiting the Tibetan Plateau’s high-altitude ecosystems revealed conserved selective regimes among five recognized species complexes. Notably, lineages experiencing upward elevational transitions exhibited significantly enhanced signatures of adaptive evolution compared to their downward-migrating counterparts [31]. After being influenced by environmental factors, not only will the genes themselves be subject to selective pressure, but their expression levels will also respond accordingly. For example, under higher temperatures, the activity of the electron transport chain in the clam, *Mytilus galloprovincialis*, was enhanced, as evidenced by increased transcript levels of ND2 and COⅠ. This contributes to the provision of ATP to meet the high energy demands of cells, thereby alleviating heat-induced energy stress and supporting energy homeostasis [32]. Mitochondrial gene expression patterns also exhibit species-specific divergence. For example, under low-temperature stress, comparative analysis revealed distinct ND5 regulation between subspecies of *Hoplobatrachus rugulosus*, with these Chinese tiger frogs showing the significant downregulation of two identical ND5 copies under low-temperature stress. By contrast, Thai tiger frogs maintained stable expression of their distinct ND5 paralogs [33]. A similar pattern was observed in *Dryophytes immaculata*, where 12 out of 13 mitochondrial genes exhibited significantly reduced transcript levels under low-temperature stress. By contrast, *Hyla zhaopingensis* demonstrated the marked upregulation of ND2 and ATP6 transcripts under low-temperature stress, a response that may be associated with its restricted distribution in low-latitude regions [34].

Previous studies have predominantly focused on mitochondrial responses to low-temperature stress [33,34,35,36], whereas research on gene expression under high-temperature stress remains limited. We designated 34 °C as the high-temperature experimental group and 25 °C as the control. This experiment selected 34 °C as the high-temperature stress condition because higher temperatures would induce near-universal upregulation of mitochondrial PCGs, rendering comparative analysis infeasible—for instance, all 13 PCGs in *Sphenomorphus incognitus* exhibited significantly elevated expression levels at 38 °C, whereas distinct differential expression patterns were observable at 34 °C. Lower temperatures, in contrast, failed to generate sufficient expression variability for meaningful statistical or functional interpretation [37]. In this study, we constructed a phylogenetic tree (using ML and BI) encompassing all four species, applied the CmC versus M2a-rel model to test for selection pressure on mitochondrial PCGs across the studied species, and quantified mitochondrial PCG expression levels under high-temperature conditions. And our aims were (1) to perform selection pressure analysis across four skink species (*Plestiodon capito*, *Plestiodon chinensis*, *Sphenomorphus indicus*, and *Scincella modesta*) to identify positively selected mitochondrial genes, (2) to evaluate whether species-specific expression-level differences of these genes exist under high-temperature stress and to provide rational explanations for such divergence, and (3) to further analyze interspecific mitochondrial expression variations to summarize whether skink species from different latitudes exhibit distinct thermal adaptation strategies in response to elevated temperatures.

## 2. Materials and Methods

### 2.1. Temperature Preferences, Distribution Range, and Grouping Strategies of Sampled Species

**Figure 1:**
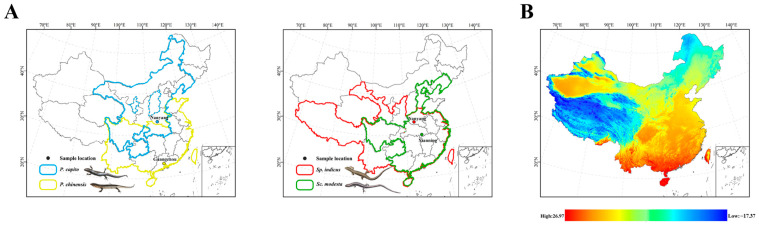
(**A**) Collection sites and geographical distribution by province (China) for *P. capito*, *P. chinensis*, *Sp. indicus*, and *Sc. modesta*. Solid lines represent provinces where the species have been observed, whereas dashed lines indicate provinces where they have not been observed. Different colors represent different species. Blue represents *P. capito*, with collection sites located in Nanyang, Henan (33°03′ N, 112°29′ E). Yellow represents *P. chinensis*, with collection sites in Guangzhou, Guangdong (23°10′ N, 113°17′ E). Red represents *Sp. indicus*, with collection sites coinciding with those of *P. capito*. Green represents *Sc. modesta*, with collection sites in Xianning, Hubei (29°34′ N, 114°29′ E). (**B**) Map of the average temperature in May in China, where red indicates a high temperature, and blue indicates a low temperature. (https://www.worldclim.org/data, accessed on 6 November 2024). ArcMap v10.8 (Esri, Redlands, CA, USA) was used to make the maps for this study.

The Scincidae family (Reptilia: Squamata), known as skinks, is rich in diversity and has a wide distribution. Indeed, this family is estimated to comprise approximately one-four of the overall skink species diversity [38]. *Plestiodon capito* is primarily distributed in the northern regions of China (Figure 1A). It mainly inhabits mountainous areas with dense vegetation and is active from late April to early October. This species is most active at temperatures of 25 °C and may seek shade to avoid the heat when temperatures exceed 30 °C [39]. *Plestiodon chinensis* is primarily distributed in southern China (Figure 1A) and has also been observed in Vietnam [40]. This species mainly inhabits low-altitude plain areas [41]. With the continuous increase in environmental temperature, its locomotor ability also increases, reaching a watershed at 34 °C [42]. *Sphenomorphus indicus* is primarily distributed in southern China and has also been observed in Sikkim, Myanmar, and Thailand. It is also found in Chinese provinces with higher latitudes, such as Gansu and Shanxi provinces (Figure 1A). The thermal preference temperature for *Sp. indicus* is 25.7 °C, making the environmental temperatures in the morning and evening more suitable for these skinks than those at noon [43]. *Scincella modesta* has a wide distribution, with populations spanning tropical and temperate regions, for example, Henan and Guangdong provinces (Figure 1A). A temperature range of 23 °C to 25 °C is considered optimal for its physiological activities. The species exhibits behavior of seeking shade when temperatures exceed 28 °C, and temperatures below 8 °C can induce hibernation [35].

Specifically, we established two comparative groups. The first group was defined within the genus *Plestiodon*, comprising *P. capito*, which is primarily distributed in high-latitude regions of northern China, and *P. chinensis* from low-latitude areas, with *P. capito*’s collection sites located at significantly higher latitudes than those of *P. chinensis*. The second group included two Sphenomorphinae species from distinct genera (*Scincella* and *Sphenomorphus*): *Sc. modesta* and *Sp. indicus*. These species partially overlap in geographic ranges, but *Sp. indicus* was collected from more northern sites, whereas *Sc. modesta* was sampled from southern locations near 30°N.

### 2.2. Sample Collection, Acclimation, and High-Temperature Stress

During late April to early May 2023, adult males of four skink species—*P. capito* (33°03′ N 112°29′ E, Nanyang, Henan), *P. chinensis* (23°10′ N 113°17′ E, Guangzhou, Guangdong), *Sp. indicus* (33°03′ N 112°29′ E, Nanyang, Henan), and *Sc. modesta* (29°34′ N 114°29′ E, Xianning, Hubei)—were collected across central and southern China (Figure 1A). We exclusively selected individuals of male to minimize experimental variability, as significant intersexual differences (e.g., sex-biased expression in the GH/IGF network [44] and hormonally regulated gene expression patterns [45]) could introduce confounding effects that compromise result reliability. Skinks of each species were collected, transported to the laboratory, and housed in identical plastic incubators (120 × 90 × 110 cm) under controlled conditions for one week acclimation prior to experiments. After acclimation, 20 skinks of appropriate size were randomly selected from each species to serve as experimental materials, with 10 individuals assigned to the control group (25 °C) and 10 to the high-temperature group (34 °C). The duration of the experiment was controlled at 24 h.

### 2.3. DNA Extraction and Sequencing

To mitigate potential interspecific variation interference in *RT*-qPCR analysis, mitochondrial sequence data were obtained for each species, and the COI gene was sequenced for 20 individuals per species. Genomic DNA was isolated from tail tissues using an Ezup Column Animal Genomic DNA Purification Kit (Sangon Biotech, Shanghai, China) following standard protocols. High-quality extractions (>25 µg/mL) underwent NGS (Illumina HiSeq 2000, PE150) by BGI Tech. (Shenzhen, China). Raw reads were quality-filtered through fastQC v0.11.6 prior to mitogenome assembly.

### 2.4. Mitochondrial Genome Assembly and Annotation

Sequencing reads were *de novo* assembled into complete mitogenomes using NOVOPlasty v4.2 [46] and GetOrganelle v1.7.1 [47]. The tRNA positions were determined using the Galaxy Europe version 23.1 platform (https://usegalaxy.eu/, accessed on 27 November 2023). The annotation of 13 PCGs, 2 rRNAs, and the non-coding control region across all four species was validated via manual curation with Mega v7.0 [48] and SnapGene Viewer v6.2.2.

### 2.5. Phylogenetic Analyses

A phylogenetic tree was constructed using 13 PCGs from 31 mitochondrial genomes, including the mitochondrial genomes of *P. capito*, *P. chinensis*, *Sp. indicus*, and *Sc. modesta*, as well as 27 mitochondrial genomes obtained from NCBI [35,49,50,51,52,53,54,55]. *Heloderma suspectum*, *Lepidophyma flavimaculatum*, *Smaug warreni*, and *Varanus salvator* [56,57,58] were used as outgroups (Table S1).

All of the above sequences were imported into PhyloSuite v1.2.3 [59] for sequence extraction. Subsequently, MAFFT v7.475 [60] was utilized to align the nucleotides of the 13 PCGs. Sequence conservation was assessed using Gblocks v0.91b [61], followed by concatenation via the built-in sequence module. A codon saturation analysis of the third positional sites performed in DAMBE v7.3.32 [62] confirmed no saturation, allowing the inclusion of all three codon positions for phylogenetic reconstruction. Optimal partitioning strategies and substitution models (Table S2) were identified through PartitionFinder v2.2.1 [63]. BI was implemented in MrBayes v3.2 [64], with partitioned data, running 10 million generations (tree sampling interval: 1000 generations), terminating at average standard deviation <0.01 to calculate posterior probabilities. ML analysis utilized RAxML-NG v1.2 [65], conducting a total of 1000 runs and utilizing a bootstrap value of 100 to assess the robustness of the ML tree.

### 2.6. Selective Pressure Analyses

The calculation of the ratio of *dN/dS* was conducted using EasyCodeML v1.41 [66] in the context of Maximum Likelihood phylogenetic analysis. The *ω* ratio is a widely utilized measure to assess the selective pressures acting on genes. In the analysis, the CmC was employed to accommodate divergence by estimating separate *ω* values for two or more clades. The M2a_rel model was used as a null model, which is derived from CmC by applying a single nonboundary constraint such that *ω_2_* = *ω_3_* [67]. Each species under study was individually considered as the foreground branch, with the remaining branches serving as the background. This approach enabled us to employ the computation of LRT *p* values and parameter estimates for the 13 PCGs. Site-specific divergence across clades was assessed via LRT comparing the CmC model against the M2a_rel null model.

### 2.7. RNA Extraction and cDNA Synthesis

For each species under study, we randomly selected four samples from both the control (25 °C) and high-temperature (34 °C) groups. A longitudinal incision was meticulously made along the ventral aspect of the abdomen. The liver was then carefully removed and positioned in a 1.5 mL microcentrifuge tube containing an RNA-free environment, ensuring optimal preservation of RNA integrity for subsequent molecular analysis. The liver was chosen as the tissue of study due to its high mitochondrial content and its role as a primary organ for energy metabolism in the body, resulting in significant expression levels [33,68]. Tissue samples were rapidly frozen in liquid nitrogen and subsequently stored at −80 °C. The total RNA was isolated from all 32 samples using the Animal Tissue Total RNA Extraction Kit (Forgene Company, Chengdu, China), with RNA integrity assessed by visualizing distinct 28S/18S rRNA bands on agarose gels. To eliminate genomic DNA contamination, RNA underwent DNase treatment at 42 °C for 2 min using the PrimeScript™ RT Reagent Kit (Takara, Japan). cDNA synthesis was then performed with the same kit under the following conditions: 37 °C for 15 min (reverse transcription), 85 °C for 5 s (enzyme inactivation), and final hold at 4 °C.

### 2.8. RT-qPCR Primer Design and Reaction

Leveraging the mitochondrial gene sequences that were acquired for each species under study, *RT*-qPCR primers were designed using Primer Premier v6.0 (Premier Biosoft International, Palo Alto, CA, USA). The *β-actin* gene was selected as the endogenous control based on its stable expression across differing temperature conditions [69]. The primer sequences for the *β-actin* gene amplification were crafted as follows: the forward primer sequence was GATCTGGCATCACACTTTCT, and the reverse primer was GTGACACCATCACCAGA [70]. Our selection of primers (Table S3) was informed by the outcomes of *RT*-qPCR reactions. Utilizing the EASY Dilution method, cDNA obtained from each sample was subjected to serial dilution to attain five discrete concentration gradients, specifically 10^−1^, 10^−2^, 10^−3^, 10^−4^, and 10^−5^. To ensure precision, we conducted three technical replicates for each primer pair to evaluate the gene expression levels. For quantification of the 13 PCGs’ transcript levels, we deployed the StepOnePlus™ Real-Time PCR System, a product of Life Technologies (Carlsbad, CA, USA). Except for the cDNA template, all other components of the reaction system were first prepared in EP tubes, vortexed, and thoroughly mixed. The thermal cycling protocol included an initial denaturation at 95 °C for 30 s, followed by 40 cycles of denaturation (95 °C, 5 s) and annealing/extension (55 °C, 30 s).

### 2.9. RT-qPCR Data Analysis

The transcript quantification of 13 mitochondrial PCGs was determined via cycle threshold (Ct) values, defined as the amplification cycles required for fluorescent signals to cross the detection threshold. Gene expression levels were computed using the 2^(−ΔΔCt) method, where ΔCt = (Ct_target_ − Ct*_β-actin_*). Data from four biological replicates were aggregated and expressed as mean ± standard error (SE). Inter-group comparisons were conducted using independent-sample *t*-tests in SPSS v21.0 (SPSS, Inc., Chicago, IL, USA) [71] with graphical outputs generated using Origin v8.0 [72]. Comparative expression profiles were additionally visualized through constructed heatmaps.

## 3. Results

### 3.1. Phylogenetic Relationships and Selective Pressure Analysis

**Figure 2:**
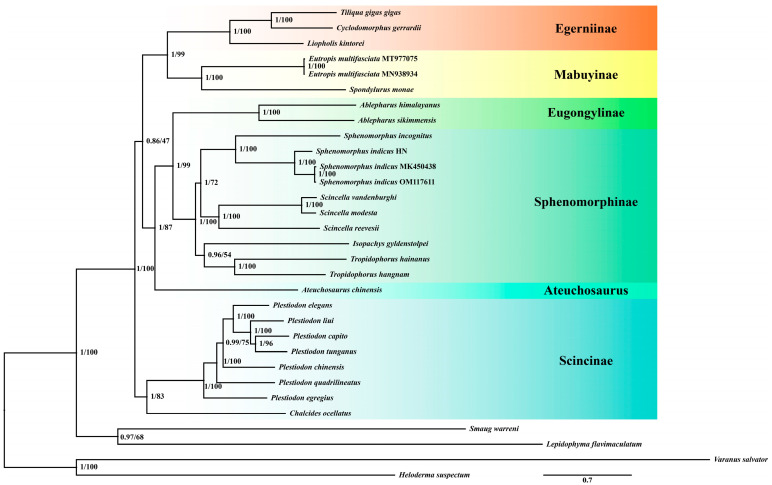
A phylogenetic tree was constructed using 13 mitochondrial PCGs from 27 species of Scincidae and 4 outgroup species using BI and ML methods. The numbers at the nodes represent the support values for those nodes, with the first number indicating the posterior probability from the BI tree and the second number representing the bootstrap value from the ML tree. Species grouped under the same color on the right side of the tree belong to the same taxonomic unit. The species *Sp. indicus* used in this study corresponds to *Sphenomorphus indicus* HN as depicted in the figure.

Based on 31 species and 13 mitochondrial PCGs, we generated phylogenetic trees using both Bayesian Inference (BI) and Maximum Likelihood (ML) methods (Figure 2). The identical topological structures of these two trees indicated that the phylogenetic relationships obtained are reliable. The resulting monophyletic groups, Egerniinae, Mabuyinae, Eugonglinae, Sphenomorphinae, and Scincinae all have high support values. Furthermore, these groups can be divided into three clades: Egerniinae and Mabuyinae form one clade, Eugonglinae and Sphenomorphinae form another clade, and Scincinae stands as a separate clade.

Using the CmC versus M2a_rel model, the ND4 gene in *P. capito* was identified as being under purifying selection (*ω* < 1), with a strong significant Likelihood Ratio Test (LRT) *p* < 0.01. In *P. chinensis*, both the ND2 and ND5 genes were identified as under purifying selection (*ω* < 1), with the ND2 gene showing a strong significant *p* < 0.01, and the ND5 gene showing a significant *p* < 0.05. In *Sp. indicus*, the ND6 gene was confirmed to be under positive selection (*ω* > 1), with a strong significant *p* < 0.01. In *Sc. modesta*, the COI and ND4 genes were suggested to be under purifying selection (*ω* < 1), with significance *p* < 0.05 (Table 1).

### 3.2. Effect of High-Temperature Stress on Transcript Levels of Mitochondrial PCGs

**Figure 3:**
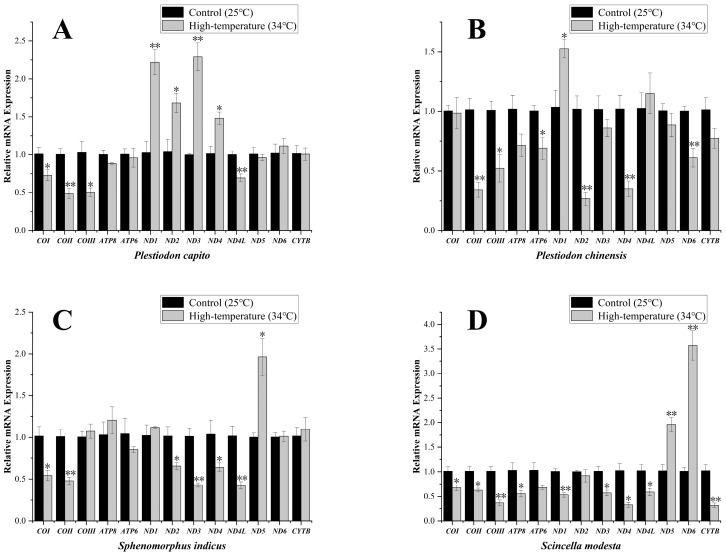
Mitochondrial-related mRNA expression in *Plestiodon capito* (**A**), *Plestiodon chinensis* (**B**), *Sphenomorphus indicus* (**C**), and *Scincella modesta* (**D**) under high-temperature stress. The expression levels of 13 mitochondrial PCGs under control conditions (25 °C) and high-temperature stress (34 °C). The *x*–axis indicates the individual genes, and the *y*-axis denotes the relative mRNA expression levels. Standard Error (SE) is used as the error bars. An asterisk “*” signifies a significant difference (*p* < 0.05), whereas “**” signifies a strong significant difference (*p* < 0.01). The expression levels were standardized relative to the *β-actin* gene as the internal control gene.

*Plestiodon capito*, inhabiting mid-to-high latitude regions, exhibited significant variation in the expression levels of four mitochondrial PCGs, the ND1, ND2, ND3, and ND4 genes showing a strong significant upregulation, to values of 2.22 ± 0.17, 1.68 ± 0.13, 2.29 ± 0.18, and 1.48 ± 0.08, respectively. By contrast, the transcript levels of several other genes were significantly downregulated, specifically for COI, COII, COIII, and ND4L genes, to values of 0.73 ± 0.07, 0.49 ± 0.07, 0.50 ± 0.06, and 0.69 ± 0.05, respectively (Figure 3A). In the low-latitude *P. chinensis*, only ND1 gene showed an upregulation of transcript levels, to values of 1.53 ± 0.08. The transcript levels of most other mitochondrial PCGs were significantly downregulated. Specifically, COII, COIII, ND2, ND4, ND6, and ATP6 genes, fell to values of 0.34 ± 0.06, 0.52 ± 0.12, 0.27 ± 0.06, 0.35 ± 0.06, 0.61 ± 0.08, and 0.69 ± 0.09, respectively (Figure 3B).

**Figure 4:**
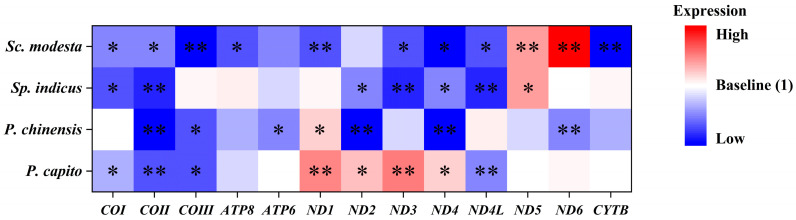
Integrating the expression levels of 13 PCGs from *P. capito*, *P. chinensis*, *Sp. indicus*, and *Sc. modesta*. The data are presented in the form of a heatmap. Red signifies high expression levels, blue signifies low expression levels, and white was used as the baseline to represent expression levels that are consistent with the control group. Asterisks denote significant differences in expression levels relative to the control group: “*” signifies *p* < 0.05, and “**” signifies *p* < 0.01.

The transcript levels of mitochondrial PCGs in *Sp. indicus*, which primarily inhabits mid-to-high latitude regions, showed changes only in the ND5 gene and showed a strong significant upregulation to values of 1.96 ± 0.22 (Figure 3C). By contrast, six significantly downregulated mitochondrial PCGs were identified. Specifically, COI, COII, ND2, ND3, ND4, and ND4L expression decreased to values of 0.54 ± 0.06, 0.48 ± 0.05, 0.66 ± 0.04, 0.43 ± 0.02, 0.64 ± 0.05, and 0.43 ± 0.04, respectively. In *Sc. modesta*, which is found at lower latitude collection sites, the ND5 and ND6 genes showed strong significant upregulation, to values of 1.96 ± 0.14, and 3.57 ± 0.30, respectively (Figure 3D). By contrast, the transcript levels of other genes were significantly downregulated, including COI, COII, COIII, ATP8, ND1, ND3, ND4, ND4L, and CYTB genes, to values of 0.68 ± 0.05, 0.63 ± 0.04, 0.37 ± 0.06, 0.56 ± 0.06, 0.53 ± 0.04, 0.57 ± 0.06, 0.33 ± 0.05, 0.59 ± 0.07, and 0.32 ± 0.05, respectively. To enable direct visual comparison of expression levels, we constructed heatmaps and annotated statistically significant genes with asterisks to highlight their differential expression patterns (Figure 4).

## 4. Discussion

### 4.1. Selective Pressure Analysis

Established through phylogenetic tree development, we conducted a selection pressure analysis to further explore the possible reasons. The phylogenetic relationships of the Scincidae based on mitochondrial PCGs are largely consistent with previous findings, ensuring the accuracy of subsequent studies [73,74,75]. By employing the CmC versus M2a_rel model, with each of the four species as the foreground branch, it was found that the ND4 gene in *P. capito* underwent purifying selection. In *P. chinensis*, the ND2 and ND5 genes experienced purifying selection. For *Sc. modesta*, both the COI and ND4 genes underwent purifying selection. Notably, the ND6 gene in *Sp. indicus* experienced positive selection, yet the expression level of this gene exhibited no significant variation relative to the control group.

Overall, we have noted the unique characteristics of the ND6 gene in *Sp. indicus* and undertook efforts to gather additional evidence, aiming to elucidate the underlying causes of this variation and its potential implications for mitochondrial function and thermal adaptation. The expression product of the ND6 gene, as a key subunit of Complex I (NADH dehydrogenase), plays an important role. Complex I, the largest protein in the respiratory chain (comprising 41 subunits), transfers electrons from NADH to ubiquinone while coupling this redox reaction to proton pumping across the inner mitochondrial membrane. This coupling mechanism drives the proton gradient required for ATP synthesis [76,77]. This also includes the subunits encoded by the mitochondrial ND1-6 and ND4L genes [78,79]. The ND6 gene has been proven to play an irreplaceable role in Complex I, located on the membrane arm of Complex I, and is involved in the proton translocation process [80,81]. The detection of positive selection in the ND6 gene implies that there may be changes in the structure or proton transport efficiency of Complex I in *Sp. indicus*. It was also observed that, in the second comparative group, the upregulated gene in *Sp. indicus* was only ND5, whereas in *Sc. modesta*, the upregulated genes included both ND5 and ND6. We speculate that this may be because the ND6 gene in *Sp. indicus* has undergone positive selection, with amino acid changes to meet the selection of the thermal environment. This can lead to changes in metabolic pathways, which may render the ND6 gene as no longer involved in the mechanisms under high temperature. The positive selection of the ND6 in skinks has been reported in a related study [49]. Additionally, a separate study revealed that within the genus *Sphenomorphus*, the ND5 gene of *Sphenomorphus incognitus* exhibited significant upregulation under 34 °C thermal stress [37]. Similar to *Sp. incognitus*, the ancestors of *Sp. indicus* likely exhibited significant upregulation of only the ND5 gene under thermal environmental temperatures, suggesting a conserved thermoadaptive mechanism.

Similar to human mitochondrial transcription, in skinks the expression of ND6 mRNA and eight tRNAs is driven by the Light-Strand Promoter (LSP), while the 12 mRNAs, two rRNAs, and remaining tRNAs are regulated by the Heavy-Strand Promoter (HSP). These two promoters drive transcription in opposite directions [82]. The mitochondrial transcription factor A (TFAM) binds to the mid-region (upstream) of both LSP and HSP, subsequently recruiting transcription factor B2 mitochondrial (TFB2M) and mitochondrial RNA polymerase (mt-RNAP) to initiate transcription [83]. The LSP terminates upstream of rRNA genes, whereas the HSP terminates within the D-loop region [82]. In our study, the differential gene expression observed between *Sp. indicus* and *Sc. modesta* was directly correlated with the differential activation of LSP and HSP. Notably, among the 12 HSP-driven mRNAs, only the ND5 gene exhibited significant upregulation, while the remaining mRNAs underwent degradation—a phenomenon whose underlying mechanism remains unclear. The LSP-mediated expression of the ND6 gene may primarily stem from differential regulation by key mitochondrial regulators. This phenomenon may be associated with peroxisome proliferator-activated receptor coactivator 1α (PGC-1α) and nuclear respiratory factors 1 and 2 (NRF-1/2), which are key regulators of the antioxidant stress response [84].

In the entire selection pressure analysis, we observed that purifying selection predominantly occurred on the ND genes, specifically ND2, ND4, and ND5. This suggests that the Complex I subunit genes are essential for cellular function, serving an indispensable role in the proton translocation process of the electron transport chain. The process of purifying selection acts to remove detrimental mutations, allowing these genes to preserve their original functions. In the ND2 and ND4 genes, *P. capito* showed significant upregulation, whereas in *P. chinensis* significant downregulation appeared. This is associated with the purifying selection on the ND2 and ND4 genes. Temperature has been described as the most significant environmental factor affecting genotypic variation among species living at different latitudes [85]. A strong relationship between environmental temperature and pressures selection on Complex I subunit genes has been described in many studies [30,86,87]. This is consistent with our observed results.

### 4.2. Analysis of Mitochondrial Genome Expression in High-Temperature Stress

In this study, we took into account various factors that influence the precise regulation of mitochondrial genomes, including the distribution range of species from external sources, differences in latitude, and phylogenetic relationships derived from genetics [88,89]. These factors can reveal the energy allocation strategies and thermal tolerance of organisms. By considering external evidence and differences in mitochondrial gene expression levels, we aim to explain the potential reasons behind mitochondrial energy allocation strategies and gene expression differences between these two comparative groups.

Our experiment did not employ extreme high-temperature stress, such as subjecting the skinks to 40 °C, but rather we used 34 °C, a temperature that may cause discomfort and potentially prompt an adjustment in mitochondrial expression levels. The 34 °C temperature was also a temperature that the four species of skinks, collected for this study, frequently encounter in their natural environment. Furthermore, as latitude decreases, the number of days per year that approach or exceed 34 °C gradually increases (Figure 1B). In the first comparative group, within the genus *Plestiodon*, *P. capito*, which has a geographical distribution and collection sites from higher latitudes, is stressed at high temperatures for a shorter period. By contrast, *P. chinensis*, which has a geographical distribution and collection sites from lower latitudes, is correspondingly stressed at temperatures exceeding or nearing 34 °C for a significantly longer period. In our experiment, six genes in *P. chinensis*, including Complex I, Complex IV, and Complex V subunit genes, showed a down regulation in transcript levels, whereas in *P. capito* only four genes exhibited a decrease, including Complex I and Complex IV subunit genes. Relative to the control group, *P. chinensis* exhibited a higher quantity of downregulated genes, with mitochondrial gene transcript levels being significantly lower. This suggests that *P. chinensis* is actively downregulating multiple mitochondrial PCGs, leading to a reduction in the quantity of various mitochondrial proteins, including those in Complex I, Complex IV (cytochrome c oxidase), and Complex V (ATP synthase), as a response to high-temperature environments. Under high-temperature conditions, mitochondria tend to engage in reverse electron transport [89], making complex I a primary producer of ROS (reactive oxygen species) [90,91,92]. ROS, as highly reactive chemical molecules, can damage cellular membranes and disrupt normal cellular physiological and molecular structures [93,94,95]. By actively reducing the amount of Complex I, *P. chinensis* can decrease the production of ROS, thereby mitigating oxidative damage potential and actively coping with a high-temperature environment.

*P. capito* exhibited the upregulation of four genes, specifically the Complex I subunits, a greater number than observed in the lower-latitude congener *P. chinensis*. This indicates that, under high-temperature stress, *P. capito* adopts a different strategy from *P. chinensis*. High-temperature environments induce mitochondrial proton leakage, reduce the effective P/O ratio (phosphorylation efficiency per oxygen consumed), and directly impair respiratory complex activity. Collectively, these effects diminish ATP synthesis efficiency and lower cellular energy reserves [96,97]. To compensate, *P. capito* upregulates genes that sustain ATP production capacity. This adaptation may support antioxidant defense systems (e.g., SOD synthesis), counteracting ROS surges induced by both thermal stress and Complex I activity [98,99,100]. The results indicate that, under high-temperature conditions, species from lower latitudes may opt to reduce their metabolic rate to cope with prolonged exposure to heat. This strategy has been confirmed in numerous studies, demonstrating that organisms can extend their survival time in adverse environments by suppressing their metabolic rates and thus decreasing their energy requirements. This indirectly suggests that animals would then exhibit stronger heat tolerance [35,36,101]. By contrast, species from mid-to-high latitudes that have fewer opportunities to experience high-temperatures are more likely to increase the expression levels of certain genes directly. This suggests that they may be in a state of thermal stress, actively upregulating gene expression as a response to high-temperature challenges [37].

In another comparative group, we observed a similar phenomenon. *Sc. modesta*, which is from a lower latitude, showed transcript levels of nine downregulated genes, including Complex I, Complex Ⅲ, Complex IV, and Complex V subunit genes, whereas *Sp. indicus*, from a higher latitude, showed transcript levels of only six downregulated genes, including Complex I and Complex IV subunit genes. Overall, the downregulation of mitochondrial gene transcript levels in *Sc. modesta* was more pronounced, akin to that observed in *P. chinensis*. This suggests that *Sc. modesta* may have adopted the same coping strategy, reducing its mitochondrial energy metabolic levels to maintain homeostasis in a more prolonged high-temperature environment. In terms of upregulated genes, *Sc. modesta*, which is from a lower latitude, actually showed the upregulation of two genes, ND5 and ND6, as compared to *Sp. indicus*, which only upregulated ND5.

The same conclusion, that low-latitude populations will have stronger heat adaptation capabilities, has also been reported in studies of other animals. For example, the distribution of clade A of *Engraulis encrasicolus* is correlated with latitude, with abundance increasing at lower latitudes, whereas clade B showed the opposite pattern. This is related to the different patterns of positive and negative selection acting on the CYTB gene, hence allowing varying capacities for heat adaptation [85]. In a study of *Drosophila melanogaster* [17], all haplotypes obtained were divided into two major haplogroups, one that was dominant at lower latitudes and the other at higher latitudes, with the abundance of both haplogroups showing a linear relationship with latitude. Using high-temperature experiments, it was directly observed that the haplogroup from lower latitudes had stronger heat tolerance. This may be related to its unique SNP types, higher GC content, and codon bias resulting in rarer codons. The expression levels of Complex I subunit genes detected in the haplogroup from lower latitudes were lower compared to those from higher latitudes, consistent with our observed data. Compared to other complex genes, genetic variation in Complex I subunit genes may have a more profound impact on mitochondrial energy metabolism and the organism’s own strategy selection [102,103]. The differences in gene expression patterns between skinks from low and high latitudes are also mainly concentrated on Complex I subunit genes. Relatively speaking, Complex IV contains genes with the lowest *dN/dS* levels, and these mitochondrial genes are subject to stronger selective pressures [104]. This is consistent with our results, where the COI, COII, and COIII genes of the four skink species were almost all downregulated compared to the control group, indicating a high degree of conservation of Complex IV subunit genes.

In summary, we propose a preliminary model of two distinct thermal adaptation strategies in skinks: (1) low-latitude species may downregulate mitochondrial gene expression to mitigate oxidative stress, providing a survival advantage in enduring prolonged high-temperature conditions prevalent. (2) Higher-latitude species, conversely, may upregulate mitochondrial gene expression to actively counteract oxidative stress, offering a tactical advantage for coping with short-term thermal stress characteristic of temperate regions with seasonal temperature fluctuations. This divergence in gene regulation aligns with their respective ecological pressures, suggesting that latitude-driven environmental variability shapes mitochondrial functional plasticity as a key mechanism for thermal adaptation.

## 5. Conclusions

In this study, we observed two distinct thermal adaptation strategies among four species of skink. Species from lower latitudes, which are stressed at high temperatures for extended periods, tend to downregulate mitochondrial genome expression levels under high-temperature, reducing the expression of many genes in order to survive continuous high-temperature conditions. For instance, *P. chinensis* and *Sc. modesta*, which are from lower latitudes, exhibited more extensive gene suppression (six and nine downregulated genes respectively) than their higher latitude counterparts. Conversely, temperate species experiencing acute thermal fluctuations preferentially upregulated key metabolic genes. This pattern was evident in *P. capito* which showed four significantly upregulated genes. Notably, whereas *Sc. modesta* exhibited the co-upregulation of ND5 and ND6 under thermal challenge, its congener *Sp. indicus* displayed selective ND5 activation. We hypothesize that positive selection on ND6 in *Sp. indicus* may have reduced its thermal responsiveness. The phylogenetic reconstruction of these taxa provided an evolutionary framework for subsequent selection pressure analyses. The high sensitivity of skink mitochondrial genes to high temperatures can reflect the climatic characteristics of their living environments, serving as an important biological indicator for assessing climate change. Future studies should conduct in-depth investigations on the ND6 gene of *Sphenomorphus* species, including thermal stress experiments on additional species to obtain expression level data and further analyze its association with phylogenetic relationships. Further analysis should focus on identifying amino acid sites under positive selection in the ND6 gene and providing detailed explanations for its altered molecular mechanisms within the respiratory chain. Expanding the research scope to encompass broader taxonomic groups is necessary to elucidate more universally applicable thermal adaptation mechanisms.

## Figures and Tables

**Table 1:** Using the CmC versus M2a_rel model, evolutionary clade model calculations were performed on 13 PCGs with the species under study as the foreground branch. Significant LRT results with *p* values < 0.05 are indicated with an asterisk “*”, and strong significant LRT results with *p* values < 0.01 are indicated with “**”. Data were filtered to include only significant results.

Species	Genes	Models	Number of Parameters	Log-Likelihood Values	Parameter Estimates	LRT *p* Values
*Plestiodon capito*	ND4	CmC M2a-rel	58 57	−17,582.82 −17,587.35	ω_0_ = 0.01, p_0_ = 0.66; ω_1_ = 1.00, p_1_ = 0.03; ω_2_ = 0.53, p_2_ = 0.31 ω_0_ = 0.01, p_0_ = 0.65; ω_1_ = 1.00, p_1_ = 0.04; ω_2_ = 0.15, p_2_ = 0.31	0.00 **
*Plestiodon chinensis*	ND2	CmC M2a-rel	58 57	−13,841.47 −13,845.21	ω_0_ = 0.01, p_0_ = 0.54; ω_1_ = 1.00, p_1_ = 0.01; ω_2_ = 0.22, p_2_ = 0.45 ω_0_ = 0.01, p_0_ = 0.54; ω_1_ = 1.00, p_1_ = 0.01; ω_2_ = 0.16, p_2_ = 0.45	0.00 **
*Plestiodon chinensis*	ND5	CmC M2a-rel	58 57	−23,658.81 −23,660.96	ω_0_ = 0.01, p_0_ = 0.62; ω_1_ = 1.00, p_1_ = 0.04; ω_2_ = 0.30, p_2_ = 0.34 ω_0_ = 0.01, p_0_ = 0.62; ω_1_ = 1.00, p_1_ = 0.03; ω_2_ = 0.19, p_2_ = 0.34	0.04 *
*Sphenomorphus indicus*	ND6	CmC M2a-rel	58 57	−6020.09 −6024.34	ω_0_ = 0.01, p_0_ = 0.70; ω_1_ = 1.00, p_1_ = 0.03; ω_2_ = 9.82, p_2_ = 0.27 ω_0_ = 0.01, p_0_ = 0.70; ω_1_ = 1.00, p_1_ = 0.03; ω_2_ = 0.12, p_2_ = 0.27	0.00 **
*Scincella modesta*	COI	CmC M2a-rel	58 57	−15,138.68 −15,141.70	ω_0_ = 0.00, p_0_ = 0.88; ω_1_ = 1.00, p_1_ = 0.00; ω_2_ = 0.47, p_2_ = 0.12 ω_0_ = 0.00, p_0_ = 0.88; ω_1_ = 1.00, p_1_ = 0.00; ω_2_ = 0.11, p_2_ = 0.12	0.01 *
*Scincella modesta*	ND4	CmC M2a-rel	58 57	−17,585.31 −17,587.35	ω_0_ = 0.01, p_0_ = 0.65; ω_1_ = 1.00, p_1_ = 0.04; ω_2_ = 0.65, p_2_ = 0.31 ω_0_ = 0.01, p_0_ = 0.65; ω_1_ = 1.00, p_1_ = 0.04; ω_2_ = 0.15, p_2_ = 0.31	0.04 *

## Data Availability

Data to support this study are available from the National Center for Biotechnology Information (https://www.ncbi.nlm.nih.gov, accessed on 24 October 2024). The accession numbers are PP946409, PP946411, PV085447, and PV085448.

## Supplementary Materials

The following supporting information can be downloaded at: https://www.mdpi.com/article/10.3390/ani15070999/s1. Table S1. The species names and accession names included in this phylogenetic tree. Table S2. The best partition schemes and nucleotide substitution models for mitochondrial data using PartitionFinder. Table S3. *RT*-qPCR primer of the 13 mitochondrial PCGs and *β-actin* in this study. Note: “NBHW” means *P. capito*, “ZHSLZ” means *P. chinensis*, “HNTTX” means *Sp. indicus*, and “DLHW” means *Sc. modesta*.
