# Supplementary material for: Differential Mitochondrial Genome Expression of Four Skink Species Under High-Temperature Stress and Selection Pressure Analyses in Scincidae
Source: Animals (Basel). 2025 Mar 30;15(7):999. doi: 10.3390/ani15070999 (PMC11988152; doi:10.3390/ani15070999)
Supplement: Supplementary file 1 [file animals-15-00999-s001.zip › Table S1.pdf]

Table S1. The species names and accession names included in this phylogenetic tree.

**Species**	**Accession No.**
Ablepharus himalayanus	MN885892
Ablepharus sikimmensis	PP439832
Ateuchosaurus chinensis	NC_057221
Chalcides ocellatus	PP571906
Cyclodomorphus gerrardii	PP571907
Eutropis multifasciata	MN938934
Eutropis multifasciata	MT977075
Heloderma suspectum	NC_008776
Isopachys gyldenstolpei	MH020638
Lepidophyma flavimaculatum	NC_008775
Liopholis kintorei	PP957932
Plestiodon capito	PP946409
Plestiodon chinensis	PV085448
Plestiodon egregius	NC_000888
Plestiodon elegans	KJ643142
Plestiodon liui	MT662111
Plestiodon quadrilineatus	PP571908
Plestiodon tunganus	MK370739
Scincella modesta	PP946411
Scincella reevesii	MN832615
Scincella vandenburghi	KU646826
Smaug warreni	NC_005962
Sphenomorphus incognitus	MH329292
Sphenomorphus indicus	MK450438
Sphenomorphus indicus	OM117611
Sphenomorphus indicus HN	PV085447
Spondylurus monae	PP291758
Tiliqua gigas gigas	PP571905
Tropidophorus hainanus	OM117612
Tropidophorus hangnam	MN977920
Varanus salvator	NC_010974
