# Supplementary material for: Differential Mitochondrial Genome Expression of Four Skink Species Under High-Temperature Stress and Selection Pressure Analyses in Scincidae
Source: Animals (Basel). 2025 Mar 30;15(7):999. doi: 10.3390/ani15070999 (PMC11988152; doi:10.3390/ani15070999)
Supplement: Supplementary file 1 [file animals-15-00999-s001.zip › Table S2.pdf]

Table S2. The best partition schemes and nucleotide substitution models for mitochondrial data using PartitionFinder.

**Subset**	**Best Model**	**Subset Partitions**
1	GTR+I+G	cytb_codon1, nad4_codon1, nad3_codon1, atp6_codon1, nad1_codon1
2	TVM+I+G	nad3_codon2, nad5_codon2, nad4L_codon2, nad2_codon2, nad4_codon2, atp6_codon2
3	HKY+I+G	nad4L_codon3, atp8_codon3, nad3_codon3, atp6_codon3
4	GTR+I+G	nad4L_codon1, nad2_codon1, nad5_codon1, atp8_codon2, atp8_codon1
5	SYM+I+G	cox1_codon1
6	TRN+I+G	cox1_codon2
7	TRN+I+G	cox1_codon3
8	SYM+I+G	cox2_codon1, cox3_codon1
9	TVM+I+G	cox3_codon2, cox2_codon2, nad1_codon2, cytb_codon2
10	HKY+I+G	cox2_codon3, cox3_codon3
11	GTR+I+G	nad2_codon3, cytb_codon3, nad1_codon3, nad5_codon3, nad4_codon3
12	HKY+G	nad6_codon2, nad6_codon1
13	HKY+I+G	nad6_codon3
