# Supplementary material for: Differential Mitochondrial Genome Expression of Four Skink Species Under High-Temperature Stress and Selection Pressure Analyses in Scincidae
Source: Animals (Basel). 2025 Mar 30;15(7):999. doi: 10.3390/ani15070999 (PMC11988152; doi:10.3390/ani15070999)
Supplement: Supplementary file 1 [file animals-15-00999-s001.zip › Table S3.pdf]

Table S3. *RT*-qPCR primer of the 13 mitochondrial PCGs and *β-actin* in this study. Note: "NBHW" means *P. capito*, "ZHSLZ" means *P. chinensis*, "HNTTX" means *Sp. indicus*, and "DLHW" means *Sc. modesta*.

Gene Name	Forward Primers (5'- 3')	Reverse Primers (5' - 3')
NBHX-*COI*	GAGCAGTGTTCGCAATTATGG	CTGAGTATCGTCGTGGTATTCC
NBHX-*COII*	ACACTTCCATGACCATGCTATC	CTCGACTTCTTGTGCGTCTATA
NBHX-*COIII*	GGCACATACCAAGGACATCATA	TCAGTAGCCGCCTAGTTCAG
NBHX-*ATP8*	GACACCCTTAGATAGCCCTACA	AAGTTCATGGTCAGTCTCATGG
NBHX-*ATP6*	CCAACCAACTCTATCTCTAGGC	GTAGAACGAATACGGCTGTAGA
NBHX-*ND1*	ACCTGACTCCTATTCTCCTCTT	CGGCTGCGTATTCTACATTGA
NBHX-*ND2*	TTCACTGACTACTTGCCTGAGT	TAGTGGTGGCTTCTGTTGCT
NBHX-*ND3*	AACAGCCCGCCTTCCATT	AGTCCAAGTGGTTGTAAGAGTG
NBHX-*ND4*	ATGACTTCCGCACCACTAATG	CACAAGGTCGGTTGCTGAG
NBHX-*ND4L*	CCATACTACCCGTGATTCTCCT	CCGTGAGTTCGAGCAGTTG
NBHX-*ND5*	AACATTGCCAATCACCACCAG	AGGCTATATGCGGCGGTTAG
NBHX-*ND6*	CAACACAACCAACACCAATTCC	TTGCGTATTCTGTGGCGTTAG
NBHX-*CYTB*	ATCGGACGAGGACTATACTACG	GGTACGGCTGATAGGAGGTT
NBHX-*β-actin*	CCTTCAACACTCCAGCCATG	TACGACCAGAGGCATACAGG
ZHSLZ-*COI*	GAGCACACCACATATTCACAGT	ACAGTATTGCGGCGTCTCA
ZHSLZ-*COII*	GCTACGAATACACAGACTACGA	GCGGTGGTCTACTTCTAATAGG
ZHSLZ-*COIII*	TTACCGCACTACAAGCCATAG	CAATTAGGAAGGTTGAGCCAAT
ZHSLZ-*ATP8*	ACCCTACTACTAGCATGAACAC	GGTACTGTAAGAGGCTGAAGAT
ZHSLZ-*ATP6*	GCCTCTTTATTCGCCCACTT	GCTGCTGTTGTAGTTATGATGG
ZHSLZ-*ND1*	TACGGCTTACTACAACCAATCG	TGAGGGCTAGGAATAGGGCTA
ZHSLZ-*ND2*	GCACAACCGCAACAATAATAGC	TGGTGTAATGGGTAGGAGAAGG
ZHSLZ-*ND3*	ACCACCACTATTGCCATCTTAC	TGGGTCAAATCCGCATTCATAG
ZHSLZ-*ND4*	ACGGCATCATTCGCATCAC	TTGTCGCAGGCAGATTGAG
ZHSLZ-*ND4L*	CTTCACCGCACTCATCTTGTC	TCGCAGGCAGAGAATGTTAGT
ZHSLZ-*ND5*	CTCCTCCATTCAAGCACCATAG	TATAGAGCCGAGACAGAGACAG
ZHSLZ-*ND6*	TTGGTTGCTGTGGCGTCTA	CGGATATGGGTCTGCTGCTA
ZHSLZ-*CYTB*	ACTTCTTGTCCTCCTACTCCTT	AGAATGGCGTATGCGAATAAGA
ZHSLZ-*β-actin*	ACACCATCACCAGAGTCCATT	CCTTCAACACTCCAGCCATG
HNTTX-*COI*	TCCATCTCGCAGGTGTATCAT	GCGGTCTGTTAATAGCATTGTG
HNTTX-*COII*	TCTTAATTGCCCTTCCCTCACT	GCAGCAGGTCTTGTGTTGG
HNTTX-*COIII*	CCACTAACAGGAGCCATC	TTAATAGGAGGACAGCAAGG

HNTTX-*ATP8*	CACAAACACCAAACAACACAAC	TAGGCTCATGGTCAGTTTCAAG
HNTTX-*ATP6*	ACCAACCAACCATATCACTAGG	GGCGAATGAATAGGCTAATTGT
HNTTX-*ND1*	CCTTATCAAGCACCGCAGTT	GTGTGACTTCGTAGGAGATTGT
HNTTX-*ND2*	GCTCGCCTGAATTGGATTAGAA	AGGCTGCTGCTTGTGTCA
HNTTX-*ND3*	ATTGCCCTACTTCTACCT	TCTGCTCATTCTAATCCG
HNTTX-*ND4*	GCCTTACTCCTAACATTCTCA	GATAACCGCTCTGCTTGG
HNTTX-*ND4L*	GCATTCATCTTGTGTCTG	GCGAGAAGTTGCTACTAA
HNTTX-*ND5*	TGCTAATCGGATGATGGTCTTC	CCAGTCAGGCTATTGAGAGGA
HNTTX-*ND6*	CCGCCAACGCTACAGAATATG	TGTGGCATCTAATCCGTCTCC
HNTTX-*CYTB*	ATCTACACATCGGACGAGGATT	AAGGCGGTTGCTATTACAAGAA
HNTTX-*β-actin*	CATGTACGTCGCCATCCAAG	CCAGAGTCCATCACGATACCA
DLHW-*COI*	AATGAGACGCCGCAATACTATG	TACGATGTCCAGTGACGAGTT
DLHW-*COII*	CCAACACAGGACCTACAACAG	ATGAGTGGAGCACGTCTTCT
DLHW-*COIII*	GCAATCTCCGACAGCGTAT	CGTAATAGGCAGACAATCAAGA
DLHW-*ATP8*	CTGGTTTCTAATCCTCCTGCTA	GGGTTGTGTGGTGGGTTAG
DLHW-*ATP6*	GCACTTGGTGTCCGACTAAC	ATGGCAACAGCAATCTCTAGG
DLHW-*ND1*	ATGGCTCCTACTATGCTCTTGA	ATTCTGATTCGCCTTCGGTAAG
DLHW-*ND2*	CCTCCGCCACTGTGCTATT	TGCCTTGTAGGACTTCTGGTAA
DLHW-*ND3*	ACCCTATGAGTGTGGCTTCG	AGGATGATGATGGTGCTTGTTC
DLHW-*ND4*	GCCTGCCTACTAGCCTTCTTA	TGATGCGAATAATGCCGTATCC
DLHW-*ND4L*	CACACCTAGTCTCTGCCTTACT	TGGTTGAGGATGGAAGTTGGA
DLHW-*ND5*	CACTACTTCACTCAAGCACCAT	CGATACAGAGGCAGATTGTCAG
DLHW-*ND6*	CCAACCAGACACCAATAAGC	GTTCTTGGTTCGTGCTGATT
DLHW-*CYTB*	TACTACTCTTCCTCCTCCTCCT	GGATGGCGTATGCGAATAGG
DLHW-*β-actin*	ACCTTCAACACGCCAGCTA	ACACCATCACCAGAGTCCAT
